# Intrafamilial Circulation of *Tropheryma whipplei*, France

**DOI:** 10.3201/eid1806.111038

**Published:** 2012-06

**Authors:** Florence Fenollar, Alpha K. Keita, Sylvain Buffet, Didier Raoult

**Affiliations:** Université de la Méditerranée, Marseille, France

**Keywords:** Whipple disease, Tropheryma whipplei, seroprevalence, intrafamilial circulation, saliva, feces, bacteria, France

## Abstract

*Tropheryma whipplei,* which causes Whipple disease, has been detected in 4% of fecal samples from the general adult population of France. To identify *T. whipplei* within families, we conducted serologic and molecular studies, including genotyping, on saliva, feces, and serum from 74 relatives of 13 patients with classic Whipple disease, 5 with localized chronic *T. whipplei* infection, and 3 carriers. Seroprevalence was determined by Western blot and compared with 300 persons from the general population. We detected *T. whipplei* in 24 (38%) of 64 fecal samples and 7 (10%) of 70 saliva samples from relatives but found no difference between persons related by genetics and marriage. The same circulating genotype occurred significantly more often in families than in other persons. Seroprevalence was higher among relatives (23 [77%] of 30) than in the general population (143 [48%] of 300). The high prevalence of *T. whipplei* within families suggests intrafamilial circulation.

## Abstract 2

High prevalence within families might reflect a specific immune condition.

Whipple disease, a rare sporadic disease, was first considered a metabolic disease (*1*) and later suspected to be an infectious disease caused by a rare bacterium, *Tropheryma whipplei* (*2*). However, the causative bacterium is common (*3**–**5*), and the well-known and classic form of Whipple disease (characterized by periodic acid–Schiff-stained bacilli in infected small-bowel macrophages) represents only 1 rare clinical form of infection caused by *T. whipplei* (*6**,**7*). In the absence of intestinal lesions, *T. whipplei* is involved in subacute or chronic infections, such as endocarditis (*8*), encephalitis (*2*), uveitis (*9**,**10*), adenopathy (*2*), and osteoarticular infections (*2**,**11*). Recently, *T. whipplei* was reported to cause acute infections, such as pneumonia (*12**,**13*), gastroenteritis (*14**,**15*), and bacteremia (*16*). Asymptomatic carriers have been identified for whom *T. whipplei* prevalence varied widely by geography or occupation (*17**–**19*). In Europe, the prevalence of *T. whipplei* in fecal samples from the general healthy adult population is ≈1%–11% (*2**,**3*). *T. whipplei* has been detected in sewage and is more prevalent in fecal samples of sewer workers (12%–26%) than in the general population (4%) (*20**,**21*). In a study in 2 rural Senegalese villages, 44% of children 2–10 years of age carried *T. whipplei* in their feces (*4*).

*T. whipplei* genotyping has shown high genetic diversity unrelated to pathogenicity, but this diversity varies geographically between Europe and Africa (*4**,**22*). Some clones circulate in particular communities, suggesting interhuman transmissibility (*4**,**14*). Moreover, the chronic carriage of *T. whipplei* in saliva and feces suggests that the bacterium might be transmissible within the same family. This question was raised to one of us (D.R.) by a person who had chronic carriage of *T. whipplei* in his saliva (*20*) and was concerned about his family. The development of *T. whipplei* serologic assays has enabled delineation between patients with Whipple disease who lack or have weak immune responses against *T. whipplei* and asymptomatic carriers who show strong immune response to the bacterium (*23**–**25*).

To identify *T. whipplei* within families, during 2003–2011 we conducted molecular and serologic investigations on samples from the families of patients who had chronic *T. whipplei* infection and were asymptomatic carriers. We also studied *T. whipplei* seroprevalence in the population of France, which enabled us to compare the prevalence with that of the families.

## Patients, Materials, and Methods

### Study Participants

The study comprised 18 patients with *T. whipplei* infections and 3 asymptomatic carriers of *T. whipplei*. Our laboratory in Marseille, France, had previously diagnosed all the infections, and one of us (D.R.) had followed-up all the patients. Samples from patients were submitted for diagnostic purposes (*3*); samples from carriers were submitted for epidemiologic studies (*20*). Our criteria to confirm classic Whipple disease included presence of positive results by periodic acid–Schiff staining and/or specific immunohistochemical results from small-bowel biopsy specimens. In contrast, the hallmark of localized extra-intestinal infection from *T. whipplei* was absence of these typical histologic lesions (*3*). The criteria for establishing the status of carriers were lack of clinical manifestations and presence of *T. whipplei* DNA within feces or saliva (*20*).

A total of 74 family members of 13 patients (10 men) with classic Whipple disease (26–78 years of age; mean ± SD age 51.4 ± 16 years), 5 (3 women) with localized chronic *T. whipplei* infection (36–71 years of age; mean ± SD age 47.2 ± 15.7 years), and 3 (all men) chronic asymptomatic carriers (27–43 years of age; mean age 33.7 years) participated. Of the family members, 40 were female relatives. Ages of family members ranged from 2 months to 79 years (mean ± SD 38 ± 22 years). A total of 64 fecal and 70 saliva specimens were analyzed by using *T. whipplei*–specific PCR. Serum samples from 30 family members were analyzed by using *T. whipplei* serologic analysis. All patients and their families provided informed consent; parents or guardians provided consent for young children.

For the seroprevalence study, 200 serum samples from blood donors from the French National Blood Service (105 men; ages of all patients 18–65 years [mean ± SD 40.74 ± 12 years]) were analyzed by Western blot. In addition, 100 control serum samples from patients hospitalized in the University Hospitals in Marseille (55 male patients; age range of all patients 1 month–88 years [mean 55.5 years]) were also analyzed by Western blot; these samples were not taken for explicit use in this study.

The overall study was approved by the local ethics committee: Institut Fédératif de Recherche 48, Marseille (agreement no. 09–018). Data obtained from adult *T. whipplei* carriers by using the same techniques were also included for prevalence comparisons (*3**,**20*).

### Molecular Assays

For each patient for whom samples were available, ≈1 g of feces and 200 µL of saliva were submitted for DNA extraction by using the QIAamp DNA MiniKit (QIAGEN, Hilden, German), according to the manufacturer’s recommendations. Quantitative real-time PCR was performed by using a LightCycler instrument (Roche Diagnostics, Meylan, France) with the QuantiTect Probe PCR Kit as described by the manufacturer (*3*). Specimens were tested by using the Twhi3F (5′-TTGTGTATTTGGTATTAGATGAAACAG-3′) and Twhi3R (5′-CCCTACAATATGAAACAGCCTTTG-3′) primer pair and the specific TaqMan probe Twhi3 (6-FAM-5′-GGGATAGAGCAGGAGGTGTCTGTCTGG-3′-TAMRA). If a specimen tested positive in this assay, the result was confirmed by a second quantitative PCR by using the Twhi2F (5′-TGAGGATGTATCTGTGTATGGGACA-3′) and Twhi2R (5′-TCCTGTTACAAGCAGTACAAAACAAA-3′) primer set and the Twhi2 probe (6-FAM-5′-GAGAGATGGGGTGCAGGACAGGG-3′-TAMRA).

*T. whipplei* detected in the specimens was genotyped by using multispacer typing as described (*22*). Each of the 4 highly variable genomic sequences from each specimen was compared with the sequences available in GenBank and in our internal laboratory database to determine their corresponding genotype.

### Western Blot

Serologic assays were performed by using Western blot. The native and deglyclosylated *T. whipplei* extracts were prepared, resolved by using sodium dodecylsulfate–polyacrylamide gel electrophoresis, and transferred onto nitrocellulose membranes as described (*23**,**24**,**26*). The membranes were immersed at room temperature in phosphate-buffered saline supplemented with 0.2% Tween 20 and 5% nonfat dry milk (blocking buffer) for 1 h before incubation with primary serum (diluted 1:1,000 in blocking buffer) for 1 h. The membranes were washed 3× with phosphate-buffered saline–Tween 20. Immunoreactive spots were detected by incubating membranes for 1 h at room temperature with peroxidase-conjugated goat anti-human antibodies (Southern Biotech, Birmingham, AL, USA) diluted 1:1,000 in blocking buffer. The assay was performed to determine the presence of *T. whipplei*–specific IgG in the serum (Southern Biotech). Detection was performed as described (*23**,**24**,**26*). Interpretation was based in particular on the analysis of a *T. whipplei* glycoprotein of 110 kDa, that is a member of the Wnt1-inducible signaling pathway proteins, a family of *T. whipplei*–specific membrane proteins as reported (*23**,**24**,**26*).

### Statistical Analysis

Statistical analyses were performed by using Fisher exact test with Epi Info 6 (www.cdc.gov/epiinfo/Epi6/EI6dnjp.htm). Results were considered statistically significant at p<0.05.

## Results

### Molecular Analysis of Saliva and Feces from Family Members

For the 74 family members of *T. whipplei*–infected patients or chronic carriers, the following familial relationships were examined: 12 sons, 10 wives, 8 daughters, 8 mothers, 6 fathers, 4 nephews, 9 grandchildren, 5 sisters, 4 husbands, 3 daughters-in-law, 2 brothers, 1 aunt, 1 stepsister, and 1 stepbrother. Family members who were positive for *T. whipplei* were tested 0–60 months (mean ± SD 33.5 ± 20 months) after treatment of their respective family member for *T. whipplei* infection.

Overall, *T. whipplei* DNA was detected in 24 (38%) of 64 fecal specimens and 7 (10%) of 70 saliva samples (Table 1). The prevalence of *T. whipplei* in feces of family members was significantly higher than that in the general population (4 [4%] of 102, p<0.001) (*20*). *T. whipplei* prevalence was also significantly higher than in feces from patients without Whipple disease (7 [2%] of 299, p<0.001) (*3*) and sewer workers (19 [9%] of 211; p<0.001) (*20*). In addition, the prevalence of *T. whipplei* in saliva from family members was significantly higher than that from patients without Whipple disease (1 [0.3%] of 432; p<0.001) (*3*). Among families of patients with classic Whipple disease, the prevalence of *T. whipplei* in feces was 14 (31%) of 45; for saliva samples, the prevalence was 4 (8%) of 48. For family members of patients with localized *T. whipplei* infection, bacterial prevalence was 2 of 9 in feces and 1 of 12 in saliva samples. Among the families of chronic carriers, *T. whipplei* prevalence was 8 of 10 in feces and 2 of 10 in saliva. For family members of chronic carriers, the prevalence in feces was significantly higher than in any other tested population, including family members of patients who had active *T. whipplei* infections (16 [30%] of 54; p = 0.004).

**Table 1:** Results of *Tropheryma whipplei* PCR on 74 relatives from 21 families of patients with classic Whipple disease, localized *T. whipplei* chronic infection, or asymptomatic carriers, France, 2003–2011

*T. whipplei*	No. relatives (no. families)	No. female relatives	Age, all patients (mean ± SD)	No. samples PCR positive/no. tested (%)		
				Feces	Saliva	Feces or saliva
Overall	74 (21)	40	2 mo–79 y (38 y ± 22 y)	24/64 (38)	7/70 (10)	25/74 (34)
Classic Whipple disease	50 (13)	29	2 mo–79 y (38.8 y ± 22 y)	14/45 (31)	4/48 (8)	14/50 (28)
Localized infection	14 (5)	5	7–75 y (40.7 y ± 24.9 y)	2/9 (22)	1/12 (8)	3/14 (21)
Carrier	10 (3)	6	8–65 y (29.6 y ± 21.1 y)	8/10 (80)	2/10 (20)	8/10 (80)

Of the 16 persons related by marriage, 5 (31%) were positive for *T. whipplei*; of the 54 persons related by genetics, 19 (35%) were positive (p = 0.99). At the time of the study, 26 (35%) of the 74 relatives lived in the same household as the related index patient, and 48 (65%) lived elsewhere. Of the 25 relatives positive for *T. whipplei*, 9 (36%) lived in the same household as the related index patient, and 16 (64%) lived elsewhere. Of the 49 relatives negative for *T. whipplei*, 17 (35%) lived in the same household as the related index patient, and 32 (65%) lived elsewhere. Persons living in the same household as the related index patient had the same prevalence regardless of whether they were (4 [33%] of 12) or were not (5 [36%] of 14) genetically related.

*T. whipplei* DNA in feces from family members ranged from 85 to 950,000 copies/g (mean ± SD 126,865 ± 296,176 copies/g); these numbers were significantly lower (p<0.001) than those of patients with active *T. whipplei* infections (range 170–6,400,000 copies/g [mean ± SD 2,410,000 ± 2,127,392 copies/g]). *T. whipplei* DNA in saliva ranged from 50 to 5,000 copies/mL (mean ± SD 2,400 ± 2,453 copies/mL) in family members and was lower than those of *T. whipplei*–infected patients (50–12,500 copies/mL [mean ± SD 3,639 ± 4,412 copies/mL]), but this difference was not significant (p = 0.5).

### Genotyping

Genotyping data were available for 5 families in which concentrations of *T. whipplei* DNA were high (Table 2). For 3 families, bacterial genotype was consistent between the patients and their families (genotypes 1, 3, and 19). Relatives from 2 of these families lived in the same household as the related index patient, whereas the relatives of the third family lived elsewhere. In 2 families, genotypes differed. In 1 family, the patient carried genotype 1, and his son had genotype 3; in the other, the patient carried genotype 82, his 2 nieces carried genotype 3, and his sister and mother carried a new genotype (83). For these 2 families, none of the relatives for whom a genotype was available lived in the same household as the index patient.

**Table 2:** *Tropheryma whipplei* genotyping for patients and their family members, France, 2003–2011*

Study participant	PCR result	HVGS1	HVGS2	HVGS3	HVGS4	Genotype	Lived in household of index patient
Patient 1 family							
Patient 1	+	1	1	1	3	1	Index patient
Father	+	1	1	1	3	1	No
Mother	+	1	1	1	3	1	No
Patient 10 family							
Patient 10	+	8	1	2	3	19	Index patient
Husband	+	8	1	2	3	19	Yes
Son	–	NA	NA	NA	NA	NA	Yes
Daughter	–	NA	NA	NA	NA	NA	Yes
Patient 11 family							
Patient 11	+	1	6	1	1	3	Index patient
Husband	+	1	6	1	1	3	Yes
Patient 7 family							
Patient 7	+	1	1	1	3	1	Index patient
Son	+	1	6	1	1	3	No
Daughter	+	NA	NA	NA	NA	NA	No
Wife	–	NA	NA	NA	NA	NA	Yes
Carrier 3 family							
Carrier 3	+	1	1	25	3	82	Index patient
Sister	+	1	6	5	1	83	No
Mother	+	1	6	5	1	83	No
Niece 1	+	1	6	1	1	3	No
Niece 2	+	1	6	1	1	3	No
Father	–	NA	NA	NA	NA	NA	No
Nephew 3	–	NA	NA	NA	NA	NA	No

*HVGS, highly variable genomic sequences; +, positive; –, negative; NA, not available.

Overall, 52 different genotypes have been identified in France from 125 persons positive for *T. whipplei*, including family members. In the family of patient 1, genotype 1 was detected in all 3 members but in only 5 of 122 other persons; this difference was significant (p<0.001). In the family of patient 10, genotype 19 was identified in 2 of 2 members (p = 0.001) but in only 3 (2%) of the 123 other persons. In the family members of carrier 3, two of 5 persons carried a new genotype (83) that has not been previously reported (0/120) (p = 0.001). In the family of patient 11, genotype 3 was detected in 2 of the 2 members; outside of the family, it was observed in 31 (25%) of 123 other persons. This difference was not significant (p = 0.06). However, of the 31 persons with genotype 3, ten were children who previously had *T. whipplei*–associated gastroenteritis, and genotype 3 was suspected to be an epidemic clone among them (*14*).

### Western Blot Serologic Analysis

#### Seroprevalence in the General Population of France

The overall seroprevalence for blood donors 18–66 years of age was 103 (52%) of 200 (Figure, panel A). Seroprevalence for patients hospitalized in the University Hospitals was comparable (Figure, panel B). However, the seroprevalence for children <4 years of age (5 [25%] of 20) was lower than in the overall population >4 years of age (35 [44%] of 80). Although this difference was not significant (p = 0.1), the seroprevalence for children <4 years of age was significantly lower than that of blood donors (103 [52%] of 200; p = 0.02).

**Figure:**
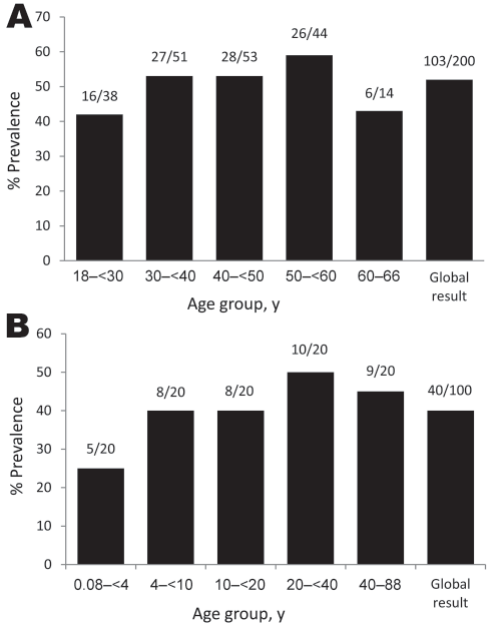
Seroprevalence of *Tropheryma whipplei* on the basis of Western blot serologic analysis of A) 200 serum samples from blood donor controls and B) 100 serum samples from patients hospitalized in University Hospitals, by age group, Marseille, France, 2003–2011.

#### Serologic Analysis for Patients and their Families

Among patients and their families tested by using Western blot analysis, 8 patients with classic Whipple disease had deficient immune response to *T. whipplei*, whereas the 2 chronic carriers showed a strong immune response (Table A1). Of the 3 patients who had localized *T. whipplei* infection, 2 had a deficient immune response, and the immune profile for 1 suggested carrier status. A positive *T. whipplei* response by Western blot occurred significantly more often in family members of patients or carriers (23 [77%] of 30) than in blood donors (103 [52%] of 200; p = 0.01) or control hospitalized patients (40 of 100; p<0.001). Of the 18 family members who were *T. whipplei* positive, 15 had a strong immune response. Of the 12 family members who were *T. whipplei* negative, 8 had a strong immune response. The 7 family members who had a deficient immune response to *T. whipplei* were from 3 different families.

## Discussion

Our data demonstrate that *T. whipplei* DNA is more prevalent in the feces and saliva of family members of patients with *T. whipplei* infection or asymptomatic carriage than in persons related by genetics or marriage. Family members were positive even if they were tested several months after their family member began antimicrobial treatment for *T. whipplei* infection. In addition, 8 of 10 persons who had close contact with chronic carriers were *T. whipplei* carriers. The comparable prevalence of this carriage among genetically related and non–genetically related relatives suggests that no genetic susceptibility exists to *T. whipplei*. The increasing prevalence of *T. whipplei* in the families seems linked to a bacterial exposure from the same source or reservoir.

The significant detection of the same bacterial genotypes in most families strongly supports the same origin of *T. whipplei* within a family. The different genotypes of *T. whipplei* within 2 families are not evidence against a common source or reservoir because *T. whipplei* has a wide heterogeneity (*4**,**22*). In 1 of the discrepant families, identification of a new genotype (83) among 2 relatives of the same family cannot clearly be linked to chance. Furthermore, the fact that relatives of 2 families with the same *T. whipplei* strain lived in the same household as the related index patient, whereas all the relatives with discrepant genotypes lived elsewhere, strongly suggest that those living together have a genetically more homogene *T. whipplei* strain than do those living elsewhere. Thus, the genotyping results and the high prevalence of *T. whipplei* in saliva and feces from family members of patients with *T. whipplei* infection or asymptomatic carriage indicate that relatives were more exposed than the general population to *T. whipplei*.

This exposition may be linked to a common source or reservoir. However, most (65%) relatives did not live in the same household and, in some instances, in the same city or region; they reported contact only during family gatherings. Also, the families did not always meet in the same place, which suggests that if the source of *T. whipplei* is common, it probably is linked to a human origin. Thus, our data strongly support the hypothesis that *T. whipplei* is transmissible between humans and is therefore contagious (Table 3). This possibility, first raised by a carrier, was initially considered unlikely. Later, the identification of clones circulating in France among children with gastroenteritis (*14*) and in western Africa (*27*) suggests this interhuman transmission. *T. whipplei* is known to be viable in feces and saliva from patients (*4**,**28*), suggesting that the bacterium might be transmitted through the fecal–oral (*2*) and oral–oral routes (*28*).

**Table 3:** Arguments for and against the intrafamilial transmission of *Tropheryma whipplei*, France, 2003–2011

Argument in favor	Argument against
Epidemiologic	
*T. whipplei* carriage is significantly more common in persons related by genetics and marriage (34%) than in the general population of France (2%–4%)	Presence of few different bacterial genotypes in some families
Significant detection of the same bacterial genotypes in most families	
Most persons did not live in the same household and had contact only during family gatherings in different places	
Microbiologic	
Positive serologic results in relatives (77%) significantly higher than in the general population of France (48%)	

Overall, the seroprevalence in family members of patients was 77%, which is higher than in that of the general population of France (≈50%). These data show that relatives of patients or chronic carriers have more frequent contact than does the general population with *T. whipplei*. In addition, seroprevalence increased with the age: seropositivity in children <4 years of age occurred less often than in older children and adult blood donors. These data suggest that persons have contact with and seroconvert against *T. whipplei* most often during childhood and that about half of the population of France has been infected with *T. whipplei*. These results are consistent with our finding that 15% of hospitalized young children tested who have gastroenteritis have high fecal loads of *T. whipplei* (*14*). However, the seroprevalence in France is lower than in rural Senegal (72.8%), where the bacterium is highly prevalent (*27*). Taken together, these data confirm that *T. whipplei* is extremely common in our environment.

Our data highlight the role of host factors in Whipple disease. For example, for patient 1, the same genotype caused asymptomatic infections in the parents who had a strong immune response to *T. whipplei* but caused Whipple disease in their child who had a deficient immune response. The lack of detectable antibodies in serum indicates a defect in the immune response. The role of immunosuppression has been documented in the worsening of Whipple disease (*2**,**29*). The overall data from the serology of the patients and their families confirm that immune responses differ between patients and asymptomatic carriers (*23**–**25*). Immune reactivity is low in patients who have *T. whipplei* infections, whereas their family members who are asymptomatic carriers develop a strong immune response to *T. whipplei*. Thus, paradoxically, the deficient immune response by use of *T. whipplei* Western blot is the current tool to differentiate patients with classic Whipple disease from *T. whipplei* carriers. Finally, the 7 family members who lacked immune responses to *T. whipplei* were from the same 3 families. Of these persons, 2 children were carriers.

The high prevalence of *T. whipplei* carriage in relatives raises several questions. Follow-up of these families will help to assess the risk for reinfection in patients successfully treated and without lifelong antimicrobial prophylaxis (*30*). In these households, 2 patients with localized chronic *T. whipplei* infection were reinfected after successful therapy. The need for systematic screening of relatives to propose a specific management will be also evaluated. We can suggest for relatives who report arthralgias the detection of *T. whipplei* by testing saliva and fecal specimens. Multiple factors may be necessary to observe the evolution from acute *T. whipplei* primary infection to chronic infection. Whipple disease is probably linked to a specific immune response to *T. whipplei* because the same genotype is responsible for various clinical manifestations and Whipple disease patients do show development of other infectious diseases. Another strong argument in favor of a specific defect in the immune response is the nature of lifetime susceptibility with relapse in patients with Whipple disease (*30*). We hypothesize that, similar to herpes virus encephalitis, a specific genetic defect might be involved in the development of Whipple disease (*2**,**31**,**32*).

Understanding of the natural history of *T. whipplei* continues to gradually increase. After contamination, including interhuman transmission, patients develop acute infection and may develop specific antibodies. Depending on host factors, patients eliminate *T. whipplei* and may harbor specific antibodies; carry it chronically for at least 5 years (D. Raoult, unpub. data) while exhibiting strong immune responses; or suffer from subacute or chronic infections, including classic Whipple disease without mainly developing antibody response.

## Appendix

**Table A1:** Results of *Tropheryma whipplei* Western blot serologic analysis for 30 relatives of 2 *T. whipplei* carriers, 8 patients with classic Whipple disease, and 3 patients with localized infections and their *T. whipplei* PCR results, France, 2003–2011*

Infection status	Age, y/sex	Immune response	Relative, *T. whipplei* PCR results, and immune response													
			Relative 1			Relative 2			Relative 3			Relative 4			Relative 5	
Relationship/age, y	Feces/saliva/ immune response	Relationship/age, y	Feces/saliva/immune response	Relationship/age, y	Feces/saliva/immune response	Relationship/age, y	Feces/saliva/immune response	Relationship/age, y	Feces/saliva/immune response
Carrier																
Carrier 1	47/M	Strong	Wife/41	+/–/strong		Son 1/12	+/+/strong		Son 2/12	+/–/strong						
Carrier 2	35/M	Strong	Wife/29	+/–/strong		-										
Classic Whipple disease																
Patient 1	40/F	Deficient	Father/71	+/+/strong		Mother/70	+/–/strong									
Patient 2	58/M	Deficient	Wife/60	–/–/strong		Daughter/32	+/+/strong									
Patient 3	48/M	Deficient	Wife/51	+/–/strong		Son/22	+/–/strong									
Patient 4	35/M	Deficient	Father/NA	–/–/deficient		Mother/NA	–/–/deficient									
Patient 5	26/F	Deficient	Father/57	–/–/strong		Mother/55	+/–/strong									
Patient 6	62/M	Deficient	Wife/62	–/–/strong												
Patient 7	43/M	Deficient	Wife/45	–/–/deficient		Son/20	+/+/deficient		Daughter/18	+/–/deficient						
Patient 8	75/M	Deficient	Wife/71	NA/–/strong					-							
Patient 9	39/F	Deficient	Husband/42	+/–/strong		Brother/50	+/+/strong		Mother/76	–/–/deficient		Father/77	+/–/deficient		Sister/52	+/–/strong
Localized infection																
Patient 10	36/M	Deficient	Wife/43	NA/–/strong		Son/12	NA/+/strong									
Patient 11	71/F	Deficient	Husband/73	+/–/strong												
Patient 12	55/F	Strong	Husband/53	–/–/strong		Mother/75	–/–/strong		Half aunt/56	–/NA/strong						

*+, positive; –, negative; NA, not available. Blank cells indicate no additional relatives.
